# Sudden Sensorineural Hearing Loss in Patient Incidental to Treatment With Hyaluronic Acid Filler

**DOI:** 10.1093/asjof/ojad091

**Published:** 2023-10-18

**Authors:** Thaís Pincelli, Han Li, Joseph T Breen, Alison Bruce

## Abstract

Hyaluronic acid fillers comprise a major component of aesthetic practice with few serious adverse effects. Hearing loss has not been previously associated with hyaluronic acid filler. The authors describe a case in which a patient developed sudden sensorineural hearing loss 1 day after filler injection into the nasolabial folds. Audiogram showed moderately severe sensorineural loss, and MRI revealed no abnormalities. Despite transtympanic steroid injections and hyperbaric oxygen therapy, the hearing loss persists at the time of writing. Although no causal relationships can be drawn from this case alone, this case serves to reinforce the importance of continued vigilance for future occurrences to minimize the potential risk of this serious adverse event.

**Level of Evidence: 5:**

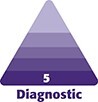

Since its approval by the FDA in 2004, soft tissue augmentation with hyaluronic acid (HA) filler has emerged as a major part of aesthetic practice.^1^ Adverse effects of dermal filler include edema or tenderness, with rarer events including arterial occlusion, infection, and long-term immune reaction.^2^ We encountered a case of acute-onset sensorineural hearing loss (SNHL) 1 day following dermal filler injection to the left nasolabial fold.

## CASE REPORT

This was a 64-year-old female who underwent a 1 cc Refyne (Galderma Laboratories, CA) filler injection into the contours of the nasolabial folds bilaterally. There was minimal bruising and swelling following the procedure. One day later, she developed a pressure-like sensation in her left ear when swallowing and muffled hearing on the left side. She returned for further evaluation 6 days later when symptoms worsened. She also reported increased sound sensitivity, particularly in settings with intense background noise, and denied vertigo or imbalance. Of note, prior to the injection, she did have a known asymmetric, left worse than right, predominantly high-frequency SNHL (pure tone average [PTA] of 22 decibels on the left vs 2 on the right). This was incidentally noted on an audiogram 7 years earlier, and an MRI revealed no retrocochlear etiology for left-sided hearing loss. She also had a history of transient benign paroxysmal positional vertigo and hypogammaglobulinemia requiring replacement immunoglobulin therapy. Her only other medication was vaginal progesterone. At the time of her otolaryngology evaluation after the injection, physical exam demonstrated patent bilateral external auditory canals, intact tympanic membranes, and aerated middle ear spaces. Her audiogram showed a flat moderately severe sensorineural loss (PTA of 50 dB), with very poor (10%) word understanding. Repeat MRI with contrast was within normal limits. The patient was given 50 mg of prednisone daily for 5 days, she underwent 3 transtympanic steroid injections, and she pursued a course of hyperbaric oxygen therapy. Unfortunately, this did not lead to significant hearing improvement (PTA 56, 24% word understanding), and she continues to follow with otolaryngology.

## DISCUSSION

The etiology of sudden SNHL is often unclear and involves multiple potential causes, including inflammation, viral infection/reactivation, and vascular alterations.^3^ HA filler-related hearing loss (HL) has only been reported once in previous literature by Henderson et al.^4^ This patient experienced immediate left-sided HL, left facial blanching, and severe pain following self-injection of HA filler to the temporal region, with imaging demonstrating occlusion of a left superficial temporal artery branch. The mechanism of HL in this case was uncertain but was hypothesized to be related to ischemia to the tympanic membrane. This mechanism was unlikely in our case given the location of the injection in the nasolabial areas and intact tympanic membrane after thorough otolaryngology (ENT) evaluation. In the context of our patient's immunoglobulin therapy, it could be postulated that HA injection triggered an autoinflammatory reaction, leading to isolated SNHL. However, despite the strong temporal correlation observed in our case, the ENT specialists considered the 2 events likely to be entirely coincidental. Her preexisting hearing asymmetry may also suggest a mechanism unrelated to filler injection. Soft-tissue fillers are the second most common nonsurgical cosmetic procedure performed annually, with 1.9 million performed in 2021.^5^ Acute SNHL occurs less frequently, but it is not infeasible that these 2 events occurred one after the other. Regardless, there are at least 2 occurrences of sudden-onset HL immediately following dermal filler injection, and this report serves to create awareness of this phenomenon. Given the ever-expanding landscape of cosmetic procedures and their associated risks, it behooves cosmetic practitioners who perform filler injection procedures to remain alert and well-informed to ensure patient safety. Conversely, it is reassuring that the ENT team did not feel the events were related and has not cautioned against future HA injections for this patient. While there is currently insufficient evidence to establish a direct causal link between HA injections and HL, continued vigilance and monitoring for future occurrences is crucial to identify and mitigate potential risks.
